# A case of an air gun bullet paranasal sinuses injury in an 11-year-old male

**DOI:** 10.1093/jscr/rjac229

**Published:** 2022-05-31

**Authors:** Alexandros Georgolios, Andrea Brestel, Adrienne Childers

**Affiliations:** 1 Otolaryngology- Head and Neck Surgery, Poplar Bluff Regional Medical Center, Poplar Bluff, MO, USA; 2 Arkansas College of Osteopathic Medicine, Fort Smith, AR, USA; 3 Department of Otolaryngology, SSM Cardinal Glennon Children’s Hospital, St. Louis, MO, USA

## Abstract

Non-powder firearms, such as BB guns, are considered safer than traditional firearms and are often marketed toward younger demographics as children’s toys. Recent advances in compressed-gas technology have drastically increased the firing power of these types of firearms, which has caused them to be more dangerous and capable of causing severe injury. We report the case of an 11-year-old male admitted for nasal injury caused by a BB gun pellet. The projectile had an unpredictable course: it pierced the skin of his left nasal sidewall, traversed through the soft tissues of the nose to the right nasal cavity violating the posterior septum, and lodged in the right posterior ethmoids adjacent to the posterior attachment of the middle turbinate. The metallic foreign object was successfully retrieved from the right nasal cavity via an endoscopic approach after minimal endoscopic dissection, guided by the preoperative radiologic imaging.

## INTRODUCTION

Non-powder guns are generally distributed unregulated in the USA. Non-powder firearms utilize the expansion of compressed air or a spring mechanism to release projectiles, and are considered safer than traditional firearms. The most common types of these guns include BB guns, pellet guns, paintball guns and air rifles. The used projectiles are plastic or metal, and vary from spherical to conical (diabolo) shape. Although the risk of injury from non-powder firearms is solidly established in the literature [1, 2], these products are ubiquitous in the USA and frequently target the pediatric population in their marketing. In the present manuscript we report the case of nasal injury in an 11-year-old male caused by a BB gun bullet, which required removal with nasal endoscopic intervention under general anesthesia. The course of the projectile was extremely unpredictable: with the entry point of the bullet in the skin of the left nasal sidewall, it traversed in an inferior-posterior direction course ending up in the posterior right nasal cavity.

## CASE REPORT

**Figure 1:**
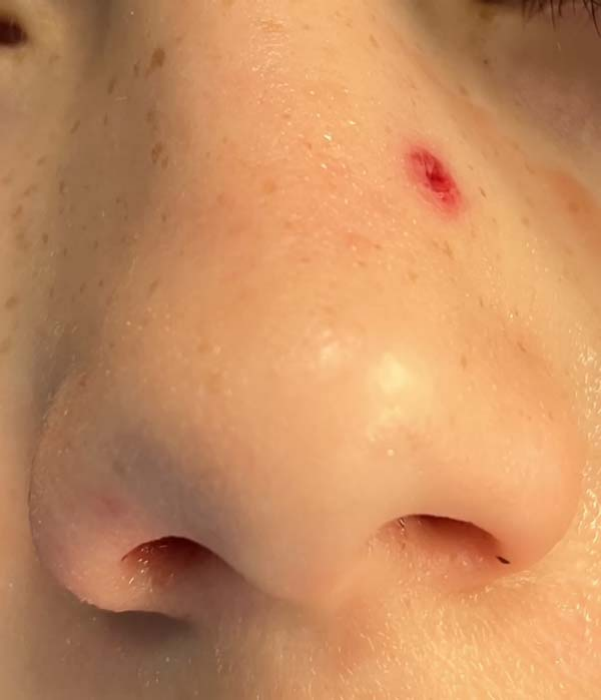
Entry wound of left nasal sidewall.

**Figure 2:**
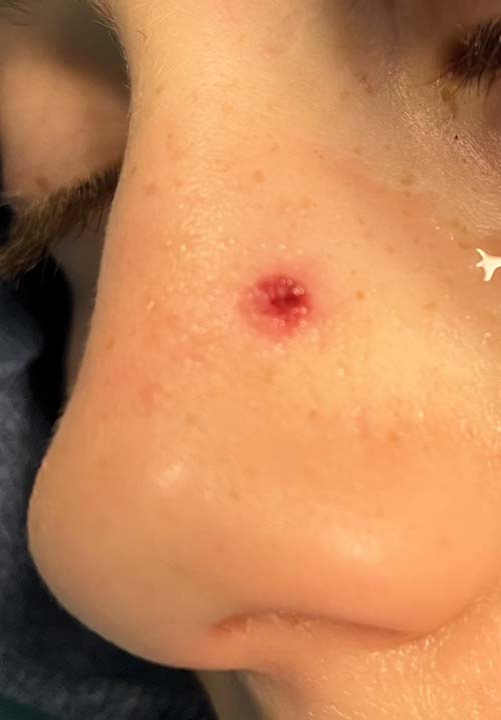
Entry wound of left nasal sidewall.

**Figure 3:**
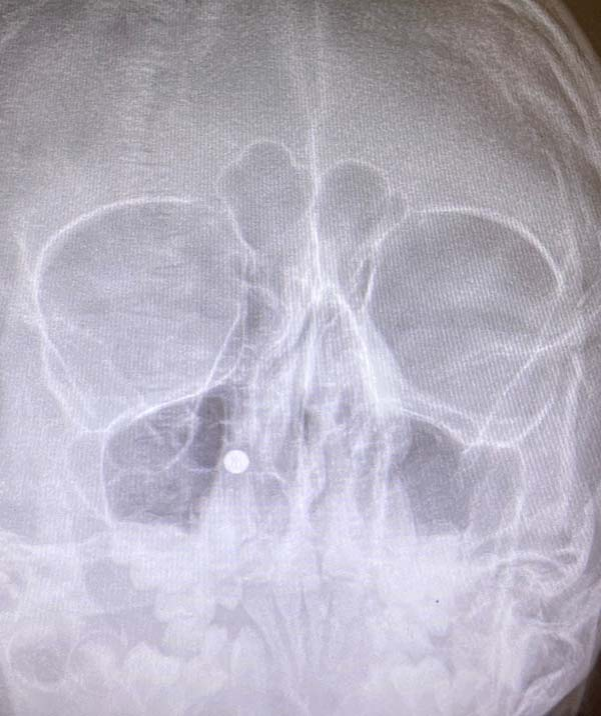
Coronal view X-ray of sinuses.

**Figure 4:**
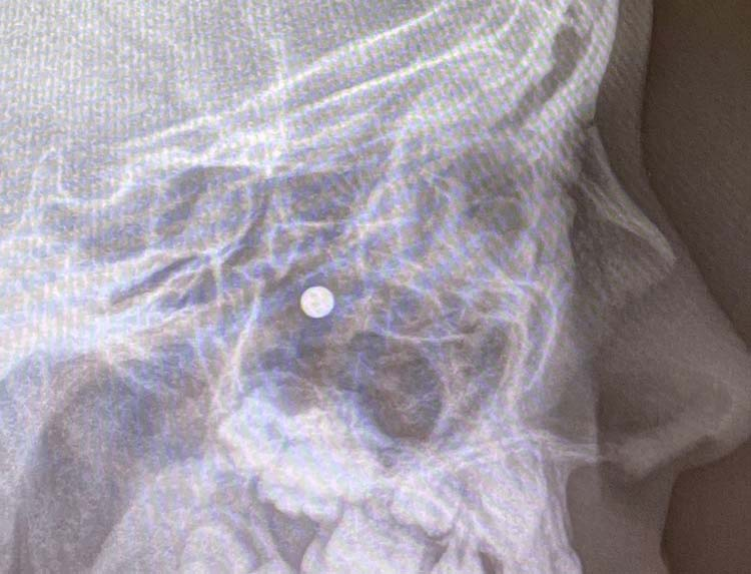
Sagittal view X-ray of sinuses.

**Figure 5:**
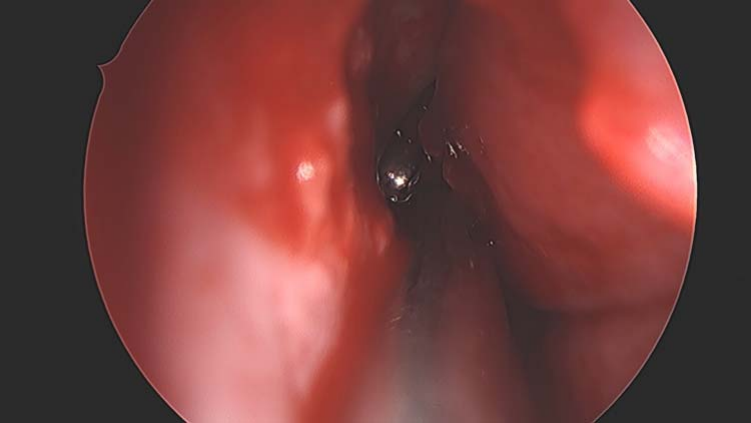
BB bullet located in the right nasal cavity.

**Figure 6:**
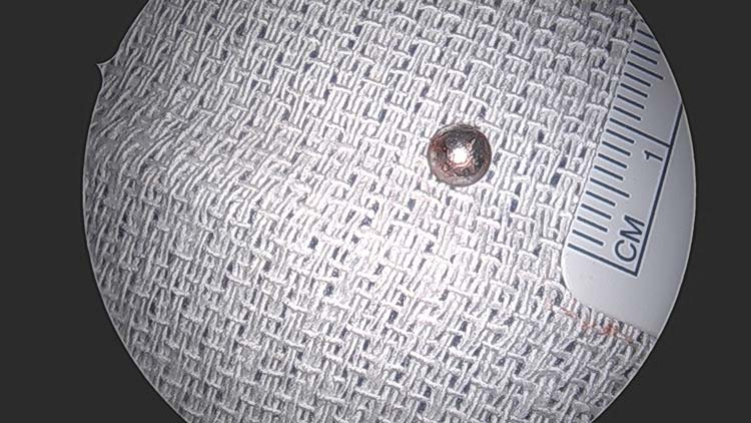
BB bullet post-extraction.

An 11 year-old male was transferred to the emergency room after being shot with a BB gun while playing. The patient’s custodian reports that he returned home with severe bleeding from bilateral nostrils which spontaneously resolved. The patient denies postnasal drip or salty taste. His medical history includes bipolar disorder and attention-deficit disorder managed with medications. On physical examination, an entry point wound is noticed in the skin of the left nasal sidewall (Figs 1 and 2). No active bleeding is noticed from anterior nasoscopy or in the posterior oropharynx exam. The rest of the head and neck examination is unremarkable. Plain X-rays performed in the emergency room confirm the presence of a metallic object in the right nasal cavity/paranasal sinuses (Figs 3 and 4). After consent is obtained, the patient is taken to the operating room and nasal endoscopy is performed. The left nasal cavity appears unremarkable. In the posterior nasal cavity, minimal bleeding and avulsed tissue is noticed after medialization of the middle turbinate. After minimal removal of tissue with pediatric Blakesley forceps, the BB bullet is visualized as lodged in the right posterior nasal cavity adjacent to the posterior attachment of the middle turbinate (Fig. 5). The bullet was then grasped with Takahashi forceps and removed after minimal endoscopic dissection (Fig. 6). Following extubation, the patient was observed for 8 h prior to discharge. During this period, no signs of recurrent epistaxis or cerebrospinal fluid rhinorrea were noticed.

## DISCUSSION

In the past, non-powder guns were traditionally spring-loaded weapons that would release their projectile at a muzzle velocity (the speed of a projectile the moment it discharges from the muzzle of a gun) at less than 350 feet per second (fps). Recent advances in compressed-gas technology have increased muzzle velocities of non-powder guns to 900–1200 fps, which approximates the muzzle velocities of modern rifles with high-velocity cartridges [3]. Case series of penetrating injuries in the head and neck caused by BB and pellet guns have been previously reported [4, 5], but there are only two reports of similar injuries with BB guns in the paranasal sinuses [6, 7]. The path of the bullet is pertinent to the unusual presentation in our case: the entry point of the bullet was observed in the skin of left nasal sidewall at the level of left upper cartilage, and plain X-ray film showed the metallic bullet was located in the right nasal cavity. In the initial evaluation under the emergency situation, this raised concerns for a laterality error and mirroring of the plain X-ray image. This was readily addressed after discussion with the radiology technicians to confirm the imaging protocol was correct. After a thorough discussion of the procedure, the patient’s custodian consented to bilateral nasal endoscopy and need for possible bilateral intervention prior to proceeding to the operating room.

Although preoperative computer tomography (CT) was considered, it was deemed redundant and we elected to avoid additional radiation to the patient. When feasible, avoiding additional radiation is reasonable for pediatric patients, especially in light of a large retrospective cohort study in the Netherlands showing a higher incidence of brain tumors in children with histories of CT scans compared with the general population [8]. It has to be noted though that the decision to skip the preoperative CT was dictated mainly by the clinical presentation (no signs of cerebrospinal fluid leak and no active epistaxis) and the plain X-ray findings suggesting the bullet was a relatively safe distance from the skull base and the orbits. Had concerns about involvement of the latter been raised, a CT would have been additionally ordered, as it also provides the opportunity for intraoperative navigation.

Finally, the trajectory of a projectile can be extremely unpredictable. In our case, the bullet passed from the skin of the left nasal sidewall in a posterior and inferior direction ending in the posterior ethmoids, just adjacent to the posterior attachment of the right middle turbinate in the territory of the sphenopalatine vessels. Surprisingly, no bleeding was noticed in the left nasal cavity during the nasal endoscopy. Penetration of the posterior septum in a full thickness manner was then identified, which was considered of no clinical significance and no specific action was taken.

## CONFLICT OF INTEREST STATEMENT

None declared.
